# Clinical characteristics and risk factors of sporadic Hepatitis E in central China

**DOI:** 10.1186/1743-422X-8-152

**Published:** 2011-04-01

**Authors:** Shujun Zhang, Jingjing Wang, Quan Yuan, Shengxiang Ge, Jun Zhang, Ningshao Xia, Deying Tian

**Affiliations:** 1 Department of Infectious Disease, Tongji Hospital, Tongji Medical College, Huazhong University of Science and Technology, Wuhan 430030, China; 2 National Institute of Diagnostics and Vaccine Development of Infectious Diseases, Xiamen University, Xiamen 361005, China

## Abstract

**Background:**

Epidemiological investigations, detections and vaccines of hepatitis E (HE) have been paid a focus of attention in prior studies, while studies on clinical features and risk factors with a large number of sporadic HE patients are scarce.

**Results:**

Sporadic HE can occur throughout the year, with the highest incidence rate in the first quarter of a year, in central of China. Of the 210 patients, 85.2% were male, and the most common clinical symptoms were jaundice (85.7%), fatigue (70.5%) and anorexia (64.8%). Total bilirubin (TBil), blood urea nitrogen (BUN), and international normalized ratio (INR) were found as major risk factors for death of HE patients. There was an overall mortality of 10%, and the mortality in the cirrhotic and non-cirrhotic group was 25% and 6.47%, respectively. Moreover, hepatitis E virus (HEV) infected patients with liver cirrhosis had a higher mortality and incidence of complications.

**Conclusions:**

TBil, BUN, and INR are major risk factors of mortality for HE. Liver cirrhosis can aggravate HE, and lead to a higher mortality. HEV infection can cause decompensation in patients with cirrhosis, as evidenced by a worsening Child-Pugh score.

## 1. Background

HE caused by HEV infection is transmitted by the fecal-oral route and generally causes an acute self-limiting illness followed by complete recovery, which is the same as hepatitis A (HA). However, the mortality of HE is higher than HA and hepatitis B (HB), especially in pregnant women (with a mortality of 20%~30%)[1]. HE is endemic in many developing countries with poor sanitation and insufficient public-health infrastructures. Nevertheless, HEV infections are reported even in developed countries in recent years, making the disease a great threat to human health. For example, a recent study reported that a seroprevalence of HEV was found among 20% of blood donors in USA and an evidence of HEV epidemic was found in Japan[2]. HEV infections have also been documented in Australia and European Union[3-6]. Besides, cases of sporadic HE in people without histories of recent travels have been reported in developed regions.

The incidence of HE is higher and higher, while mortalities in different areas are distinct. A study in India revealed that the mortality of out-break of HE was 0.07%-0.6%[7]. The mortality of in-hospital patients with acute HE had a mortality of 1%~3%[1]. Till now, most studies were focused on the epidemiological investigation, detections and vaccines, while studies on clinical features and risk factors of death for HE with a large number of patients are lacking. There are studies on the outcome of HEV infection in patients with chronic liver disease from India, Nepal, France and the UK[8-11]. It was showed that the mortality of HEV in patients with cirrhosis was 70% at 1 year[8]. However, similar reports from China are scarce.

## 2. Methods

### 2.1 Patients

This study included 210 in-hospital HE patients from Department of Infectious Disease of Wuhan Tongji Hospital from January 2007 to December 2008. HE case definition: alanine aminotransferase, ALT ≧ 2.5 × upper limits of normal (ULN) and HEV IgM positive, or a rising HEV IgG or HEV PCR positive[11]. Cirrhotic patients with sepsis, primary liver cancer, surgical obstructive jaundice, hepatorenal syndrome and those consuming alcoholic during previous 6 months were excluded from the study. The cirrhosis groups were matched for Child-Pugh score twice: the first time was 1 month before admission and the second was after admission. Each patient after discharge from the hospital was followed up 4 weekly at least for 6 months.

### 2.2 Methods

#### 2.2.1 Pathogenic Detection

Sera from each patient was tested for HEV-IgM, HEV-IgG, HAV-IgM, anti-HCV, HBsAg, HBsAb, HBeAg, HBeAb, HBAb using commercial ELISA kit ( Beijing Wantai Company).

#### 2.2.2 Reverse transcription and nested PCR for HEV[12]

HEV RNA was extracted and precipitated from 200 μl of serum samples by acid-guanidinium-phenol method (Trizol LS Reagent Invitrogen, USA). Reverse primer E5: 5'CTACACGAAACCGARAGW (R = A or G, W = A or C) was used to reverse transcription. With primer E1 (5'CTGTTTAAYCTTGCTGACAC 3'(Y = C or T)) and primer E5, the first round of amplification was completed (94℃ pre-degeneration for 5 min, 94℃ for 40s, 53℃ for 40s, 72℃ for 40s, followed by 35 cycles, 72℃ for 10 min). The amplified material was used for the second-round nested amplification with primers E2 (5'GACAGAATTGATTTCGTCG 3') and E4 (5'GTCCTAATACTRTTGGTTGT3' (R = A or G)). The length of PCR product corresponding to ORF2 sequence was 189 bp (6298nt-6486nt).

#### 2.2.3 Biochemical Tests

An automatic biochemical analyzer (Beckman L220) was used to analyze biochemical parameters, such as ALT, BUN, TBil, INR, aspartate aminotransferase (AST), albumin (ALB), total cholesterol (Tchol), lactate dehydrogenase (LDH), creatinine (Cr), prothrombin activity (PTA).

#### 2.2.4 Diagnosis of cirrhosis

Forty of 210 patients were diagnosed with liver cirrhosis before HEV infection, which was established by conventional clinical, biochemical, imaging, and endoscopic criteria[8]. The etiology of cirrhosis in patients was HB 20, Schistosomiasis 7, alcohol 3, hepatitis C 1, autoimmune hepatitis 1, alcohol plus HB 5, HB plus Schistosomiasis 1, Schistosomiasis plus alcohol 1, and HB plus Schistosomiasis and alcohol 1 case.

#### 2.2.5 Statistics

Quantitative variables were expressed as means (± SD) and compared by the Student t-test, or represented as median (25th percentile-75th percentile). Mann-Whitney U test was used to compare serum biochemical indicators and χ2 or Fisher's exact test was used to enumeration data. Odds ratio (OR) for all variables was calculated by univariate and multivariate logistic regression. All statistical calculations were performed using SPSS software 13.0.

## 3. Results

### 3.1 Etiology Detected Results

Among 210 patients, 125 were diagnosed being infected with HEV alone, 1 co-infected with hepatitis A virus (HAV), and 75 co-infected with hepatitis B virus (HBV), 2 with hepatitis C virus (HCV), 3 with cytomegalovirus (CMV), 3 with Epstein-Barr virus (EBV), 1 with CMV + EBV. Forty of the 210 patients had liver cirrhosis as well. Seventy-eight patients had detectable HEV RNA in their sera. All of them were genotype 4 which were confirmed by bidirectional sequencing and phylogenetic.

### 3.2 The Incidence Rate of HE in Different Seasons

**Figure 1:**
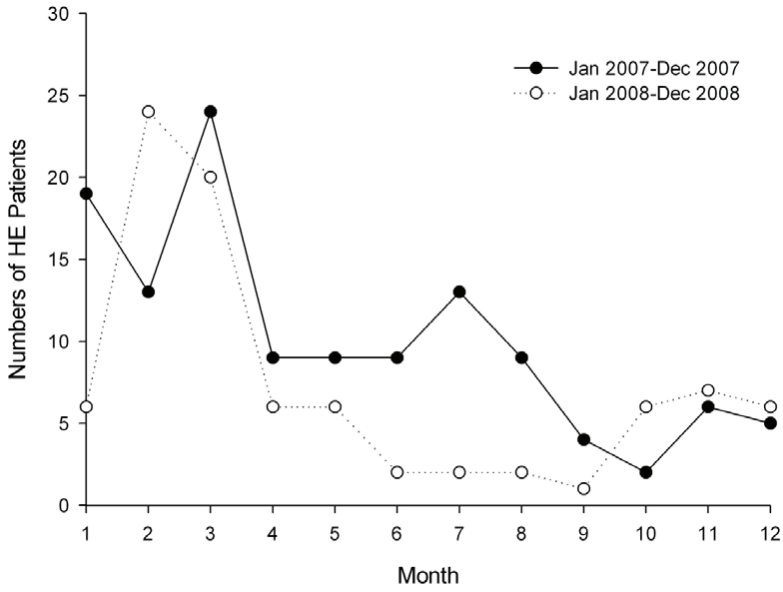
**The incidence of HE infection in different months of the year**.

It was found that 122 and 88 patients were infected with HE in 2007 and 2008, respectively. Although patients could be infected throughout a year, the incidence rate of HE was highest in the first quarter (from January to March) (Figure 1).

### 3.3 The Distribution of HE patients' Gender and Age

**Figure 2:**
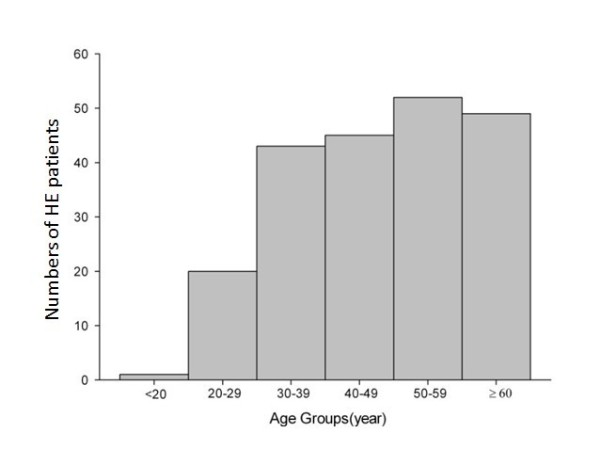
**Number of HE patients according to age group**.

Of the 210 patients, 179 cases were male (85.2%) and 31 cases were female (14.8%). The ratio of male to female was approximately 5.8:1. Ages of the patients ranged from 17 to 92 years old (48.7 ± 14.9). There was only 1 case under 20 years old (0.5%), 90% of the patients were over 30 years of age (Figure 2).

### 3.4 Clinical Symptoms and Serum Biochemistry

Jaundice, fatigue and anorexia were the most common clinical symptoms, with incidence rates of 85.7% (180/210), 70.5% (148/210), and 64.7% (136/210), respectively. Other less common symptoms were also observed, including abdominal distention (55/210, 26.2%), nausea (23/210, 11.0%), vomiting (14/210, 6.7%) and fever (16/210. 7.6%).

All patients were followed up for six months. Twenty-one patients died during this period, for a mortality of 10% (95% CI: 6.63%-14.80%). The main causes of death were upper gastrointestinal bleeding (6/21), hepatorenal syndrome (12/21), and hepatic encephalopathy(3/21). The results of clinical features and serum biochemistry were summarized in Table 1. Patients with old ages and liver cirrhosis were more likely to die than others. The level of TBil, Tchol, LDH, PTA and INR were significantly different between died patients and survivors. BUN and Cr, which reflect the renal function, were higher in the deceased group than in the survivor group.

**Table 1:** Comparison of clinical and biochemical parameters between the deceased group and the survival group

Variable	Deceased group	Survival group	*P value*
Number of patients	21	189	-
Age in years	59.67 ± 13.99	47.22 ± 13.97	0.0002
Gender(male/female)	20/1	159/30	NS
ALT(U/L)	731.00(202.00-1910.50)	1070.50(381.00-1773.00)	NS
AST(U/L)	659.00(228.10-1386.00)	551.00(189.00-1242.25)	NS
GGT(U/L)	62.00(44.00-115.50)	131.00(73.00-221.00)	0.007
TBil(μmol/L)	551.40(409.35-607.95)	163.81(69.83-275.90)	< 0.001
ALB(g/L)	29.60(26.40-31.30)	34.15(31.15-37.78)	< 0.001
ALP(U/L)	199.00(144.50-241.50)	170.00(131.00-218.00)	NS
Tchol(mmol/L)	1.33(0.98-1.86)	3.23(2.51-3.92)	< 0.001
LDH(U/L)	274.00(223.00-385.00)	200.00(151.00-261.00)	0.001
CHE(U/L)	2941.00(2060.00-4269.50)	4454.50(3021.25-5808.75)	0.003
BUN(mmol/L)	14.50(6.07-24.51)	4.44(3.64-5.61)	< 0.001
Cr(μmol/L)	124.05(70.83-253.55)	64.00(55.38-73.78)	< 0.001
PTA(%)	46.00(32.00-70.00)	92,50(71.00-112.75)	< 0.001
INR	1.66(1.35-2.19)	1.14(1.05-1.30)	< 0.001

ALT, alanine aminotransferase; AST, aspartate aminotransferase; GGT, γ-glutamyltransferase; TBil, total bilirubin; ALB, albumin; ALP, alkaline phosphatase; Tchol, total cholesterol; LDH, lactate dehydrogenase; CHE, cholinesterase; BUN, blood urea nitrogen; Cr, creatinine; PTA, prothrombin activity; INR, international normalized ratio; and NS, not significant.

From table 2 we can see that age (OR = 1.067), TBil (OR = 1.010), ALB (OR = 0.781), Tchol (OR = 0.164), LDH (OR = 1.007), BUN (OR = 1.299), Cr (OR = 1.010), PTA (OR = 0.956), and INR (OR = 6.113) were the death risk factors of HE. Multivariate analysis of death risk factors in patients with HE were shown in Table 3. TBil, BUN, and INR were major risk factors

**Table 2:** Risk factors of mortality among patients with HE by Univariate analysis

Variable	Categories	Odds Ratio	95% CI	*P value*
Age	Continuous	1.067	1.029--1.106	0.0005
Gender	Female	1		
	Male	4.173	0.538--32.343	NS
ALT	Continuous	1.000	0.999-1.000	NS
AST	Continuous	1.000	1.000-1.000	NS
GGT	Continuous	1.000	0.997-1.002	NS
TBil	Continuous	1.010	1.006-1.013	< 0.001
ALB	Continuous	0.781	0.696-0.877	< 0.001
ALP	Continuous	1.001	0.999-1.003	NS
Tchol	Continuous	0.164	0.079-0.340	< 0.001
LDH	Continuous	1.007	1.003-1.011	0.001
CHE	Continuous	1.000	0.999-1.000	0.005
BUN	Continuous	1.299	1.149-1.469	0.001
Cr	Continuous	1.010	1.003-1.016	0.003
PTA	Continuous	0.956	0.937-0.975	< 0.001
INR	Continuous	6.113	2.401-15.561	< 0.001

ALT, alanine aminotransferase; AST, aspartate aminotransferase; GGT,γ-glutamyltransferase; TBil, total bilirubin; ALB, albumin; ALP, alkaline phosphatase; Tchol, total cholesterol; LDH, lactate dehydrogenase; CHE, cholinesterase; BUN, blood urea nitrogen; Cr, creatinine; PTA, prothrombin activity; INR, international normalized ratio; and NS, not significant.

**Table 3:** Risk factors of mortality among patients with HE by Multivariate analysis

Variables	Categories	Odds Ratio	95% CI	*P *value
TBil	Continuous	1.0009	1.004-1.014	< 0.001
BUN	Continuous	1.178	1.034-1.341	0.014
INR	Continuous	9.216	1.969-43.129	0.005

TBil, total bilirubin; BUN, blood urea nitrogen; INR, international normalized ratio.

### 3.5 The Influence of Liver Cirrhosis on the Prognosis of Hepatitis E

Compared with hepatitis E infection patients without liver cirrhosis, the mortality of those with liver cirrhosis was increased (25% vs.6.47%, *P *= 0.002). The incidence of hepatic encephalopathy (6/40 vs. 8/170, p = 0.03), hepatorenal syndrome (8/40 vs. 5/170, *P *< 0.001) and spontaneous bacterial peritonitis (24/40 vs. 14/170, *P *< 0.001) were higher than HEV infected patients without cirrhosis. The difference of ALT, GGT, ALB, Tchol, PTA, INR, BUN between two groups was significant as well (table 4).

**Table 4:** Comparison of demographic, biochemical parameters as well as Complications and mortality frequencies among patients infected with genotype 4 HEV with and without liver cirrhosis

Parameter	HEV		*P value*
	
	with cirrhosis	Without cirrhosis	
Number of patients	40	170	-
Age in years	51.78 ± 14.70	48.02 ± 14.55	NS
Gender(male/female)	36/4	143/27	NS
Mortality	10(25%)	11(6.47%)	0.002
Complications			
Spontaneous peritonitis	24(60%)	14(8.24%)	< 0.001
Upper gastrointestinal hemorrhage	2(5%)	4(2.35%)	NS
Hepatorenal syndrome	8(20%)	5(2.94%)	< 0.001
Hepatic encephalopathy	6(15%)	8(4.71%)	0.03
ALT(U/L)	574.5(150.25-1612.25)	1158(478.75-1816.75)	0.02
AST(U/L)	310.5(117-1198.5)	670.5(208-1265.2)	NS
GGT(U/L)	85(52.5-114.75)	131(67-229)	0.002
TBil(umol/L)	242.6(115.6-530.3)	197.93(82.6-298.85)	NS
ALB(g/L)	31(27.65-32.98)	34.1(31.58-37.8)	< 0.001
Tchol(mmol/L)	2.17(1.71-3.27)	3.18(2.45-3.9)	< 0.001
CHE(U/L)	3014.5(2125.75-3967)	4356(3039.25-5752.25)	0.001
BUN (mmol/L)	5.5(4-9.29)	4.65(3.64-6)	0.032
Cr(umol/L)	64.9(55.35-116.6)	65.5(56.2-78.8)	NS
PTA(%)	63(42-85)	93(72.95-113.5)	< 0.001
INR	1.41(1.19-1.75)	1.13(1.04-1.29)	< 0.001

HEV, hepatitisE virus; ALT, alanine aminotransferase; AST, aspartate aminotransferase; GGT, γ-glutamyltransferase; TBil, total bilirubin; ALB, albumin; ALP, alkaline phosphatase; Tchol, total cholesterol; LDH, lactate dehydrogenase; CHE, cholinesterase; BUN, blood urea nitrogen; Cr, creatinine; PTA, prothrombin activity; INR, international normalized ratio; and NS, not significant

The 40 HE patients with cirrhosis were assessed by Child-Pugh's score on the basis of reviewing their history, clinical symptoms and laboratory data one month ago. And this was before presenting HEV infection. Thirty-two cases (80%) were at the Child-Pugh stage A, 7 cases (17.5%) at the Child-Pugh stage B, and 1 case (2.5%) at the Child-Pugh stage C, with a mean score of 5.85 ± 1.29. After being in hospital, 4 cases (10%) were at stage A, 21 cases (52.5%) at stage B, and 15 cases (37.5%) at stage C, with a mean score of 8.83 ± 1.97. Child-Pugh's score of the patients after being in hospital was significantly worse than the patients before HEV infection (5.85 ± 1.29 vs. 8.83 ± 1.97, *P *< 0.001).

## 4. Discussion

There are four genotypes of HEV, but only one serotype. Genotypes 1 and 2 exclusively infect humans, whereas genotypes 3 and 4 can also infect other animals, particularly pigs. Genotype 4 has been proven the dominant genotype in China since the year of 2000[13-16]. In this study, seventy eight (37.14%) of 210 patients had detectable HEV RNA in their sera. All patients in whom HEV RNA was isolated were infected with genotype 4, which was congruent with those from China.

Contaminated drinking water has been served as sources of several outbreaks of HE in India[17-19]. It demonstrates that the contaminated water is one important reason for outbreak of HE, therefore outbreaks of HE were found in rainy or floodwater seasons. However, in contrast with the outbreak of HE, our data illustrated that the sporadic HE infection could occur throughout a year, and exhibited obvious seasonal occurrence. The incidence of HE in the first quarter of 2007 and 2008 were much higher than that in other quarters. HE is a zoonotic disease. The strongest evidence of zoonotic transmission of HE is from Japan[20,21]. Similarly, more meat in central of China is consumed in the first quarter of a year, since people in China celebrate their most important traditional festivals (the Spring Festival and the Lantern Festival) in the first quarter. So, this may be a possible reason for the seasonal occurrence. Another reason is that the weather in central of China in the first quarter is conducive to virus multiplication and propagation.

Although HEV and HAV are similar viruses in terms of the transmission mode and clinical manifestation, 90% of the HE patients were over 30 years of age, and we saw only one case under age 20 in this study, which are rather different from the HA patients. The reason for the difference of the age distribution is could be that HEV has a much lower secondary attack rate among exposed household members compared with the stable HAV (with a secondary attack rate of 20%-50%) [22,23].

Another noticeable feature of HE is that the male patients were much more than the female patients (5.8:1). The result is in accordance with other reports. For example, a nationwide survey of the prevalence of IgG anti-HEV in qualified blood donors throughout Japan showed that prevalence of IgG anti-HEV was higher in men (3.9%) than in women (2.9%) [24]. Similar results were also reported in Bangladeshi and Taiwan[25,26]. In this study, jaundice, fatigue and anorexia were most common clinical manifestations. The mortality was 10.0% in our study, higher than the previously reported rate of 1% to 3%. This difference may be mainly due to the sample selection in this study: a considerable proportion of patients whom were hospital referraled from municipal hospitals were critically ill, leading to a high mortality in the overall sample (Tongji hospital is the largest hospital in the middle part of China).

The multivariate analysis showed that TBil, BUN, and INR were major risk factors of mortality for HE, which was useful to assess the prognosis of HE. Infection of HEV in patients on the base of chronic liver diseases could make the chronic liver disease more severe, and cause decomposition and death[27]. Patients with cirrhosis were prone to infect HEV, and the mortality of HEV infected cirrhotics at 4 weeks and 12 month was increased compared with that of non-infected cirrhotics[8]. Our study also showed that the mortality of HEV infected patients with cirrhosis was higher. And the incidence of hepatic encephalopathy, hepatorenal syndrome and spontaneous bacterial peritonitis were higher than HEV infected patients. It reveals that patients with cirrhosis would have a worse condition as well as a poor prognosis. The hepatic reserve function of cirrhotics deteriorated after HEV infection.

**In conclusion**, we found that TBil, BUN, and INR were major risk factors of mortality of HE based on 210 patients in central of China. Liver cirrhosis could make the HE more severe, and result in a higher mortality. And HEV infection could cause decompensation in patients with cirrhosis. Although the pathogenesis of HE has not been clarified due to the lack of effective cell or animal models of HEV infection, the results are helpful to provide us some ideas for clinical diagnosis, treatment and basic research of HE.

## Competing interests

The authors declare that they have no competing interests.

## Authors' contributions

SJZ conceived of the study, and participated in its design and coordination and helped to draft the manuscript. JJW participated in collecting samples and following up. QY and SXG carried out the molecular genetic studies, participated in the sequence alignment. JZ performed the statistical analysis. NSX and DYT participated in the design of the study. All authors read and approved the final manuscript.
